# Family History of Breast Cancer and Mammographic Breast Density in Premenopausal Women

**DOI:** 10.1001/jamanetworkopen.2021.48983

**Published:** 2022-02-17

**Authors:** Yunan Han, Justin Xavier Moore, Graham A. Colditz, Adetunji T. Toriola

**Affiliations:** 1 Division of Public Health Sciences, Department of Surgery, Washington University School of Medicine, St Louis, Missouri; 2 Cancer Prevention, Control, and Population Health Program, Department of Medicine, Augusta University, Augusta, Georgia; 3 Alvin J. Siteman Cancer Center at Barnes-Jewish Hospital and Washington University School of Medicine, St. Louis, Missouri

## Abstract

**Importance:**

Family history of breast cancer (FHBC) and mammographic breast density are independent risk factors for breast cancer, but the association of FHBC and mammographic breast density in premenopausal women is not well understood.

**Objectives:**

To investigate the association of FHBC and mammographic breast density in premenopausal women using both quantitative and qualitative measurements.

**Design, Setting, and Participants:**

This single-center cohort study examined 2 retrospective cohorts: a discovery set of 375 premenopausal women and a validation set of 14 040 premenopausal women. Data from women in the discovery set was collected between December 2015 and October 2016, whereas data from women in the validation set was collected between June 2010 and December 2015. Data analysis was performed between June 2018 and June 2020.

**Exposures:**

Family history of breast cancer (FHBC).

**Main Outcomes and Measures:**

The primary outcomes were mammographic breast density measured quantitatively as volumetric percent density using Volpara (discovery set) and qualitatively using BI-RADS (Breast Imaging Reporting and Data System) breast density (validation set). Multivariable regressions were performed using a log-transformed normal distribution for the discovery set and a logistic distribution for the validation set.

**Results:**

Of 14 415 premenopausal women included in the study, the discovery set and validation set had similar characteristics (discovery set with FHBC: mean [SD] age, 47.1 [5.6] years; 15 [17.2%] were Black or African American women and 64 [73.6%] were non-Hispanic White women; discovery set with no FHBC: mean [SD] age, 47.7 [4.5] years; 87 [31.6%] were Black or African American women and 178 [64.7%] were non-Hispanic White women; validation set with FHBC: mean [SD] age, 46.8 [7.3] years; 720 [33.4%] were Black or African American women and 1378 [64.0%] were non-Hispanic White women]; validation set with no FHBC: mean [SD] age, 47.5 [6.1] years; 4572 [38.5%] were Black or African American women and 6632 [55.8%] were non-Hispanic White women]). In the discovery set, participants who had FHBC were more likely to have a higher mean volumetric percent density compared with participants with no FHBC (11.1% vs 9.0%). In the multivariable-adjusted model, volumetric percent density was 25% higher (odds ratio [OR], 1.25 ;95% CI, 1.12-1.41) in women with FHBC compared with women without FHBC; and 24% higher (OR, 1.24; 95% CI, 1.10-1.40) in women who had 1 affected relative, but not significantly higher in women who had at least 2 affected relatives (OR, 1.40; 95% CI, 0.95-2.07) compared with women with no relatives affected. In the validation set, women with a positive FHBC were more likely to have dense breasts (BI-RADS 3-4) compared with women with no FHBC (BI-RADS 3: 41.1% vs 38.8%; BI-RADS 4: 10.5% vs 7.7%). In the multivariable-adjusted model, the odds of having dense breasts (BI-RADS 3-4) were 30% higher (OR, 1.30; 95% CI, 1.17-1.45) in women with FHBC compared with women without FHBC; and 29% higher (OR, 1.29; 95% CI, 1.14-1.45) in women who had 1 affected relative, but not significantly higher in women who had at least 2 affected relatives (OR, 1.38; 95% CI, 0.85-2.23) compared with women with no relatives affected.

**Conclusions and Relevance:**

In this cohort study, having an FHBC was positively associated with mammographic breast density in premenopausal women. Our findings highlight the heritable component of mammographic breast density and underscore the need to begin annual screening early in premenopausal women with a family history of breast cancer.

## Key Points

**Question:**

Is family history of breast cancer (FHBC) associated with mammographic breast density in premenopausal women?

**Findings:**

In this cohort study of 14 415 premenopausal women, volumetric percent density was significantly higher in women with FHBC compared with women without FHBC, and the odds of having dense breasts was significantly higher in women with FHBC compared with women without FHBC.

**Meaning:**

The findings highlight the heritable component of mammographic breast density and underscore the need to begin annual screening early in premenopausal women with a family history of breast cancer.

## Teaser

This cohort study investigates the association of family history of breast cancer and mammographic breast density in premenopausal women using both quantitative and qualitative measurements.

## Introduction

Women with a positive family history of breast cancer (FHBC) in a first-degree relative (mother, sister) have a 2- to 4-fold increased risk of breast cancer.^1,2,3,4^ A dense breast on a mammogram is also associated with an increased risk of breast cancer.^1,5,6^ Women with more than 75% dense area of breast have a 4- to 6-fold increased risk of breast cancer compared with women with less than 5% dense area.^7,8,9^ Premenopausal women have denser breasts than postmenopausal women as mammographic breast density decreases slowly with age.^10,11^

Mammographic breast density and breast cancer share similar genetic pathways,^12,13^ hence, we hypothesize that FHBC will be associated with a higher risk of having dense breasts, especially in premenopausal women. However, only a few studies have investigated the associations between these 2 strong risk factors for breast cancer in premenopausal with conflicting results.^14,15,16^ Two studies observed that a FHBC was positively associated with having dense breasts. They, however, did not stratify analyses by menopausal status or race, which is important because the only study in Asian women reported no association of FHBC with mammographic breast density.^16^ Furthermore, these studies evaluated mammographic breast density using Breast Imaging Reporting and Data System (BI-RADS).^14,15^ As many breast health centers begin to adopt volumetric measures of mammographic breast density in clinical practice, it is crucial to have data on the associations of FHBC with volumetric measures such as volumetric percent density. In addition, a greater understanding of the association of FHBC with mammographic breast density in premenopausal women could help identify high-risk women whose mammography screening strategies may need to be refined.

The main objectives of our study were to determine the association between FHBC and mammographic breast density in premenopausal women and to clarify whether these associations are consistent or differ by the type of mammographic breast density measure used (ie, quantitative vs qualitative measure). To accomplish this, we (1) investigated the association of FHBC with mammographic breast density using Volpara (quantitative measures); (2) validated the findings within a larger study population using BI-RADS (qualitative measures); (3) further stratified analyses by race to determine whether race modifies the associations of FHBC on mammographic breast density.

## Methods

### Study Population

The study population included 2 groups of women recruited at the Joanne Knight Breast Health Center (BHC) at Washington University School of Medicine and Siteman Cancer Center. The discovery set consisted of 383 premenopausal women recruited during routine screening mammograms in 2016. Mammographic breast density was measured quantitatively using Volpara. The validation set consisted of 14 040 premenopausal women recruited while undergoing screening and diagnostic mammograms between June 2010 and December 2015. Mammographic breast density was measured qualitatively using BI-RADS. We received study approval through the Washington University School of Medicine institutional review board, and all study participants provided informed consent. The study followed the Strengthening the Reporting of Observational Studies in Epidemiology (STROBE) reporting guideline.^17^

### Discovery Set

Among the 383 premenopausal women in the discovery set, our current study is limited to 375 women with complete data. A detailed description of the study population has been provided previously,^18,19^ and is given only briefly here. Premenopausal women who were scheduled for their annual screening mammography at the BHC were mailed study flyers by research coordinators 2 weeks to 1 month in advance. Follow-up calls were made within 1 week of the scheduled appointments to screen interested individuals and to provide further details on the study. To be eligible, participants had to be (1) premenopausal at the time of mammogram; we identified women as premenopausal if they had a regular menstrual period within the preceding 12 months, no prior history of bilateral oophorectomy, and not used menopausal hormone therapy; (2) no serious medical condition that would prevent the participant from returning for her annual mammogram in 12 months, (3) not pregnant; (4) no history of cancer, including breast cancer; (5) and no history of breast augmentation or reduction. On the day of mammographic screening, participants completed a questionnaire with detailed information on demographics, reproductive and anthropometric measures, and breast cancer-related risk factors.

Our primary outcome in the discovery set was volumetric percent density, measured using Volpara (version 1.5, Volpara Solutions, Wellington, New Zealand).^20^ We also measured dense volume, and nondense volume using Volpara. Volpara volumetric percent density values range from 0.5% to 34.5% with a typical median of around 6%.^20,21^ In comparison with BI-RADS categories, Volpara volumetric percent density values less than 3.5% correspond to BI-RADS a: almost entirely fatty; volumetric percent density at least 3.5% and less than 7.5% correspond to BI-RADS b: scattered areas of fibroglandular density; volumetric percent density at least 7.5% and less than 15.5% correspond to BI-RADS c: heterogeneously dense; volumetric percent density at least 15.5% correspond to BI-RADS d: extremely dense.^22^ (Categories a, b, c, and d correspond to BI-RADS 1, 2, 3, and 4, respectively, in the Results section.)

### Validation Set

Women scheduled for mammogram at the BHC from 2010 to 2015 were mailed study flyers 2 weeks to 1 month before their visitation. Among a total of 56 423 women who underwent routine screening with mammograms, we excluded participants if they were postmenopausal (n = 31 893) at the time of mammogram. We identified women as premenopausal using the same criteria used for the discovery set. We additionally excluded women who were currently pregnant, had a history of breast cancer, and had a history of breast augmentation or reduction (n = 3551). Lastly, participants were excluded if they had missing information regarding mammographic breast density (n = 6266) or race (n = 473). Thus, 14 040 premenopausal women were eligible for analysis.

Our outcome of interest in the validation set was BI-RADS breast density. Women were categorized into BI-RADS a: almost entirely fatty; BI-RADS b: scattered areas of fibroglandular tissue; BI-RADS c: heterogeneously dense; BI-RADS d: extremely dense.^23,24^ For analytic purposes, we dichotomized women as either having dense (heterogeneously and extremely dense breast) or nondense (almost entirely fatty and scattered fibroglandular tissue).

### Primary Exposure of Interest

Our primary exposure of interest was FHBC. We defined FHBC as participants with a first-degree relative (including only biological mother and sisters) with a history of breast cancer. Therefore, FHBC was categorized into a dichotomous variable of yes and no. We further stratified this variable among women with FHBC in mothers or sisters alone. Lastly, we analyzed the total number of affected relatives with an FHBC: 0 = participants with zero first-degree relatives with a history of breast cancer; 1 = one mother or sister with breast cancer, 2 = mother and at least one sister with a history of breast cancer.

### Other Variables

Baseline demographic variables included self-reported race (Black or African American, non-Hispanic White, and others), age (continuous), and education (less than or equal to a high school graduate, post-high school training or some college, college graduate, and post-graduate degree). Education was obtained only in the discovery set. Health behaviors included ever use of oral contraceptives (yes or no) and current alcohol use (yes or no). Lastly, breast cancer-related risk factors included in this study were age at menarche, parity, age at first childbirth, and current body mass index (BMI). We calculated BMI as current weight in kilograms divided by current height in meters squared.

### Statistical Analysis

Descriptive statistics are presented as mean and standard deviation (SD) for continuous variables, and number and relative frequencies (%) for categorical variables. We examined differences in participants’ characteristics by FHBC using ANOVA for continuous variables and χ^2^ test for categorical variables. We performed a series of analytic multivariable models to test the associations between FHBC with (1) quantitative measures of mammographic breast density (using volumetric percent density, dense volume, and non-dense volume) and (2) and qualitative measures of mammographic breast density (using BI-RADS categories). Quantitative measures were natural log-transformed to ensure the normality of the residuals in all regression models. Two multivariable linear regression models were used to evaluate the associations between FHBC and log-transformed volumetric percent density, dense volume, and nondense volume. Model 1 was adjusted for current age (continuous, years) and BMI (continuous). Model 2 was adjusted for current age (continuous, years), BMI (continuous), parity (0, 1, 2, ≥3), race (Black or African American, non-Hispanic White, or others), age at menarche (continuous, years), and alcohol consumption (yes or no). β coefficients and 95% CIs from the regression models were evaluated and back-transformed for easier interpretation. In our second series of models, we performed logistic regression analyses with BI-RADS breast density as the dependent variable and FHBC as the primary explanatory variable. We adjusted these models similar to the aforementioned linear regression models for quantitative mammographic breast density measures. Results from logistic regression were presented as odds ratios (ORs) and associated 95% CIs. To determine if there were any race-specific differences in the effects of FHBC on mammographic breast density, we a priori decided to perform all linear and logistic regression models further stratified by race (Black or African American, or non-Hispanic White).

The American College of Radiology BI-RADS released the 5th edition in 2013,^25^ thus we performed sensitivity analysis stratifying our validation cohort into 2 subcohorts based on when they had their mammograms: pre-2013 (before January 1, 2013) and post-2013 (January 1, 2013, and after). All the analyses were performed using SAS software version 9.4 (SAS Institute) from June 2018 to June 2020. All tests were 2-tailed, and statistical significance was considered as *P* < .05.

## Results

### Characteristics of Participants

Of 14 415 premenopausal women included in the study, the discovery set and validation set had similar characteristics (discovery set with FHBC: mean [SD] age, 47.1 [5.6] years; 15 [17.2%] were Black or African American women and 64 [73.6%] were non-Hispanic White women; discovery set with no FHBC: mean [SD] age, 47.7 [4.5] years; 87 [31.6%] were Black or African American women and 178 [64.7%] were non-Hispanic White women; validation set with FHBC: mean [SD] age, 46.8 [7.3] years; 720 [33.4%] were Black or African American women and 1378 [64.0%] were non-Hispanic White women; validation set with no FHBC: mean [SD] age, 47.5 [6.1] years; 4572 [38.5%] were Black or African American women and 6632 [55.8%] were non-Hispanic White women]) (Table 1).

**Table 1.** Participant Characteristics of Premenopausal Women Recruited During Annual Mammogram at the Joanne Knight Breast Health Center by Family History of Breast Cancer Status, Stratified by Study Cohorts

Characteristics	No. (%)						
	Discovery set (n = 375)				Validation set (n = 14 040)		
	FHBC (n = 87)	No FHBC (n = 275)	Unknown FHBC (n = 13)	*P* value ^a^	FHBC (n = 2153)	No FHBC (n = 11 887)	*P* value^a^
Race							
Black/African American	15 (17.2)	87 (31.6)	7 (53.9)	.007	720 (33.4)	4572 (38.5)	<.001
Non-Hispanic White	64 (73.6)	178 (64.7)	5 (38.5)		1378 (64.0)	6632 (55.8)	
Other^b^	8 (9.2)	10 (3.6)	1 (7.7)		55 (2.6)	683 (5.8)	
Current age, mean (SD), y	47.1 (5.6)	47.7 (4.5)	47.5 (5.8)	.69	46.8 (7.3)	47.5 (6.1)	<.001
Age, mean (SD), y							
At menarche^c^	13.0 (1.8)	12.8 (2.3)	12.6 (1.8)	.67	12.7 (1.7)	12.8 (1.7)	.007
At first childbirth^d^	26.4 (5.8)	25.9 (6.3)	23.8 (3.0)	.49	24.7 (6.2)	24.4 (6.4)	.06
Current BMI, mean (SD)	31.0 (9.6)	30.4 (7.5)	34.5 (9.1)	.20	29.7 (8.0)	29.9 (8.1)	.35
Parity^e^							
0	23 (26.4)	42 (15.4)	5 (38.5)	.006	298 (13.8)	1468 (12.4)	.04
1	8 (9.2)	55 (20.2)	4 (30.8)		375 (17.4)	2134 (18.0)	
2	38 (43.7)	100 (36.8)	1 (7.7)		709 (32.9)	3676 (30.9)	
≥3	18 (20.7)	75 (27.6)	3 (23.1)		588 (27.3)	3557 (29.9)	
Ever breastfed^f^	53 (60.9)	157 (57.1)	6 (46.2)	.05	NA	NA	NA
Ever oral contraceptive use	77 (88.5)	246 (89.5)	10 (76.9)	.37	1563 (72.6)	8438 (71.0)	.13
Education^g^							
High school orless	7 (8.1)	27 (9.9)	2 (15.4)	.41	NA	NA	NA
Post-high school or less than college	15 (17.2)	56 (20.4)	4 (30.8)		NA	NA	NA
College graduate	30 (34.5)	110 (40.2)	4 (30.8)		NA	NA	NA
Postgraduate	35 (40.2)	81 (29.6)	2 (15.4)		NA	NA	NA
Current alcohol use^h^	62 (71.3)	173 (62.9)	9 (69.2)	.37	1188 (55.2)	5970 (50.2)	<.001
Quantitative mammographic breast density							
Volumetric percent density, %	11.1 (7.1)	9.0 (6.1)	8.3 (7.9)	.02	NA	NA	NA
Dense volume, cm^3^	92.0 (47.6)	77.8 (41.3)	61.5 (18.1)	.007	NA	NA	NA
Nondense volume, cm^3^	1076.1 (818.5)	1073.2 (717.1)	1144.9 (829.6)	.94	NA	NA	NA
Qualitative mammographic breast density							
Extremely dense	NA	NA	NA	NA	227 (10.5)	912 (7.7)	<.001
Heterogeneously dense	NA	NA	NA	NA	885 (41.1)	4611 (38.8)	
Scattered fibroglandular	NA	NA	NA	NA	891 (41.4)	5343 (45.0)	
Almost entirely fatty	NA	NA	NA	NA	150 (7.0)	1021 (8.6)	

Abbreviations: BMI, body mass index calculated as weight in kilograms divided by height in meters squared; FHBC, family history of breast cancer; NA, not applicable.

^a^ The *P* value was calculated using ANOVA for continuous variables and χ^2^ test for categorical variables.

^b^ Other races and ethnicities included Hispanic, Asian, American Indians/Alaska Native, and Native Hawaiian/Pacific Islander.

^c^ In cohort 1, no FHBC (n = 273).

^d^ In cohort 1, FHBC (n = 64), no FHBC (n = 230), unknown FHBC (n = 8).

^e^ In cohort 1, no FHBC (n = 272).

^f^ In cohort 1, FHBC (n = 64), no FHBC (n = 233), unknown FHBC (n = 8).

^g^ In cohort 1, no FHBC (n = 274), unknown FHBC (n = 12).

^h^ In cohort 1, no FHBC (n = 274).

#### Discovery Set

Among the 375 eligible participants in the discovery set, 87 women (23.2%) had a FHBC (Table 1). Current age, age at menarche, age at first birth, BMI, breastfeeding, oral contraceptives, education, and alcohol were similar in participants across FHBC categories. Compared with participants with no FHBC, participants who had a FHBC were more likely to be non-Hispanic White women (73.6% vs 64.7%), more likely to be nulliparous (26.4% vs 15.4%), more likely to have a higher mean volumetric percent density (11.1% vs 9.0%), and more likely to have a higher mean dense volume (92.0 cm^3^ vs 77.8 cm^3^).

#### Validation Set

Among the 14 040 eligible participants in the validation set, 2153 (15.3%) had a FHBC (Table 1). Similarly, compared with women with no FHBC, women with a positive FHBC were more likely to be non-Hispanic White (64.0% vs 55.8%), more likely to be nulliparous (13.8% vs 12.4%), more likely to use alcohol currently (55.2% vs 50.2%), and more likely to have dense breasts (BI-RADS 3 and 4) (BI-RADS 3: 41.1% vs 38.8%; BI-RADS 4:10.5% vs 7.7%).

### Associations Between FHBC and Mammographic Breast Density

#### Discovery Set

The associations between FHBC and volumetric percent density are presented in Table 2. In the multivariable-adjusted model (model 2), volumetric percent density was 25% higher (OR, 1.25; 95% CI, 1.12-1.41; *P* < .001) among women who had a FHBC than women with no FHBC. Women with a positive FHBC in mothers alone had a 31% higher volumetric percent density (OR, 1.31; 95% CI, 1.15-1.48; *P* < .001) compared with women with had no FHBC in mothers. Having a positive FHBC in sisters alone was not associated with higher volumetric percent density, likely due to the small number of women who had a positive FHBC in sisters alone (n = 26). Additionally, we analyzed the number of first-degree relatives with FHBC and volumetric percent density. Volumetric percent density was 24% higher (OR, 1.24; 95% CI, 1.10-1.40) in women who had 1 affected relative, but not statistically significantly higher in women who had at least 2 affected relatives compared with women with no relatives affected (OR, 1.40; 95% CI, 0.95-2.07).

**Table 2.** Associations of Family History of Breast Cancer With Volumetric Percent Density, and BI-RADS Breast Density Categories

Characteristics	Discovery set (n = 375)			Validation set (n = 14 040)		
	Volumetric percent density (%)^a^			Dense breast (BI-RADS)^b^		
	No.	exp^β^ (95% CI)	*P* value^c^	No.^d^	Odds ratio (95% CI)	*P* value^e^
FHBC						
Model 1^f^						
No	275	1 [Reference]	NA	5523	1 [Reference]	<.001
Yes	87	1.25 (1.12-1.40)	<.001	1112	1.29 (1.15-1.43)	
Unknown	13	1.01 (0.77-1.31)	.96	NA	NA	NA
Model 2^g^						
No	275	1 [Reference]	NA	5523	1 [Reference]	<.001
Yes	87	1.25 (1.12-1.41)	<.001	1112	1.30 (1.17-1.45)	
Unknown	13	1.00 (0.76-1.30)	.99	NA	NA	NA
FHBC in mothers alone						
Model 1^f^						
No	301	1 [Reference]	NA	4922	1 [Reference]	<.001
Yes	67	1.31 (1.15-1.48)	<.001	769	1.27 (1.12-1.45)	
Unknown	7	1.03 (0.72-1.47)	.87	945	1.33 (1.18-1.49)	
Model 2^g^						
No	301	1 [Reference]	NA	4922	1 [Reference]	<.001
Yes	67	1.31 (1.15-1.48)	<.001	769	1.28 (1.12-1.46)	
Unknown	7	1.03 (0.72-1.47)	.86	945	1.32 (1.18-1.49)	
FHBC in sisters alone						
Model 1^f^						
No	339	1 [Reference]	NA	4919	1 [Reference]	<.001
Yes	26	1.08 (0.89-1.31)	.45	256	1.32 (1.08-1.61)	
Unknown	10	1.04 (0.77-1.41)	.80	1460	1.30 (1.18-1.44)	
Model 2^g^						
No	339	1 [Reference]	NA	4919	1 [Reference]	<.001
Yes	26	1.07 (0.88-1.30)	.48	256	1.34 (1.09-1.64)	
Unknown	10	1.03 (0.76-1.40)	.85	1460	1.30 (1.17-1.43)	
No. of affected relatives with FHBC						
Model 1^f^						
0	275	1 [Reference]	NA	4916	1 [Reference]	<.001
1	81	1.24 (1.10-1.40)	<.001	936	1.28 (1.14-1.44)	
≥2	6	1.38 (0.94-2.03)	.10	44	1.38 (0.85-2.23)	
Unknown	13	1.01 (0.77-1.31)	.96	739	1.34 (1.17-1.52)	
Model 2^g^						
0	275	1 [Reference]	NA	4916	1 [Reference]	<.001
1	81	1.24 (1.10-1.40)	<.001	936	1.29 (1.14-1.45)	
≥2	6	1.40 (0.95-2.07)	.09	44	1.38 (0.85-2.23)	
Unknown	13	1.00 (0.76-1.31)	.99	739	1.32 (1.16-1.51)	

Abbreviations: BI-RADS, The Breast Imaging, Reporting and Data System; exp, exponentiated; FHBC, family history of breast cancer; NA, not applicable.

^a^ Continuous mammographic breast density measurements were log transformed in the analysis. The β coefficients were back transformed and exponentiated for easier interpretation. Effect measures represent proportion for percentage difference.

^b^ Dense breast defined as BI-RADS 3 and 4. Nondense breast (reference) defined as BI-RADS 1 and 2. Logistic regression examines odds of having dense breasts.

^c^ *P* value derived from linear regression.

^d^ Represents the proportion of women with dense breasts within the exposure strata (ie, among those with or without a family history of breast cancer).

^e^ Type III χ^2^ *P* values derived from logistic regression.

^f^ Model 1 was adjusted for current age (continuous, years) and BMI (continuous).

^g^ Model 2 was adjusted for current age (continuous, years), BMI (continuous), parity (0, 1, 2, ≥3), race (Black or African American/non-Hispanic White/other), age at menarche (continuous, years), and alcohol consumption (yes/no).

We observed similar positive associations between FHBC and dense volume (eTable 1 in the Supplement). Dense volume was 16% higher (OR, 1.16; 95% CI, 1.04-1.29; *P* < .001) in women with a positive FHBC compared with women with no FHBC in the multivariable-adjusted model. There were no associations between FHBC and nondense volume (eTable 1 in the Supplement).

#### Validation Set

The odds of having dense breasts were 30% higher (OR, 1.30; 95% CI, 1.17-1.45) among women with a positive FHBC compared with women with no FHBC in the multivariable-adjusted model. The odds of having dense breasts were 28% higher (OR, 1.28; 95% CI, 1.12-1.46) among women with FHBC in mothers alone compared with women with no FHBC in mothers alone (Table 2). The odds of having dense breasts were 34% higher (OR, 1.34; 95% CI, 1.09-1.64) among women with FHBC in sisters alone compared with women with no FHBC in sisters alone. The odds of having dense breasts were 29% higher (OR, 1.29; 95% CI, 1.14-1.45) among women with 1 family member with FHBC, but were not statistically significantly higher among women with 2 or more family members with FHBC (OR, 1.38; 95% CI, 0.85-2.23).

There was no effect modification by race (eTable 2 in the Supplement). Associations of FHBC with mammographic breast density were identical among non-Hispanic White and Black or African American women. In sensitivity analysis, the associations of FHBC and mammographic breast density were similar among women who had their mammograms before and after implementation of BI-RADS 5th edition in 2013 (eTable 3 in the Supplement).

## Discussion

We found that having a positive FHBC was associated with higher mammographic breast density in premenopausal women using both quantitative and qualitative measures. In the multivariable adjusted model, women with a positive FHBC had a 25% higher volumetric percent density than women with no FHBC among the discovery set. Findings were validated in a larger data set of 14 040 women: women with a positive FHBC had a 30% increased odds of having dense breasts compared with women with no FHBC. Findings were similar for non-Hispanic White and Black or African American women.

Studies on the association of FHBC with mammographic breast density in premenopausal women have yielded inconsistent results. Yang et al^16^ reported no associations between FHBC and mammographic breast density. Several reasons may explain the discordance of our study findings. First, only 4.8% of their study participants had a positive FHBC,^16^ compared with 23.2% in our discovery set, and 15.3% in our validation set. Second, they only adjusted their analyses for age, whereas we comprehensively adjusted for many important variables, hence, residual confounding could have accounted for the lack of associations they observed. Third, differences in participants’ race and ethnicity^26^ may also account because their study population only included Asian women whereas our study population was diverse and consisted mainly of non-Hispanic White and Black or African American women. Furthermore, the mean age of their study participants was 43.2 years, compared with 47.6 years in our discovery set and 47.4 years in our validation set.

Our findings complement findings in a previous study, which also reported positive associations between FHBC and mammographic breast density. Ziv et al^14^ reported that women with categories of BI-RADS 3 and 4 breast density were 70% more likely to have a positive FHBC among 6146 women with mixed pre-and postmenopausal statuses (BI-RADS 3: OR, 1.70; 95% CI, 1.19-2.40; BI-RADS 4: OR, 1.70; 95% CI, 1.05-2.71). Our study participants’ characteristics, however, are different from the study by Ziv et al.^14^. We focused on premenopausal women, this is important because hormone changes after menopause cause the breast tissue to become less dense.^27^ Moreover, we excluded women with any history of cancer, whereas their analysis included women with personal breast cancer history.^14^ Hence, our work provides new information about the associations between FHBC and mammographic breast density among premenopausal women.

### Strengths and Limitations

Our study has several strengths. First, our discovery cohort recruited participants among women attending annual routine screening mammography at the Joanne Knight BHC, which enhances generalizability. At the same time, we used the larger validation set with 14 040 participants to validate our findings in the discovery set, enhancing reproducibility. Second, our large sample size in the validation set allows us to thoroughly assess the associations among women with FHBC in mothers alone or sisters alone. In addition, our validation data set included women who had their mammograms pre- and postimplementation of the BI-RADS 5th edition, and as a result we were able to explore whether differential classifications of BI-RADS were associated with odds of having dense breasts. Lastly, to the best of our knowledge, this is the first study to investigate the associations of FHBC with volumetric measures of mammographic breast density.

Our study also has some limitations. First, the prevalence of participants with a positive FHBC history in 2 study sets were higher (23.2% in discovery set; 15.3% in validation set) than the National Health Interview Survey for premenopausal women (aged 40 to 49 years: 8.4%). This is because women who had a positive FHBC are more likely to undergo annual screening.^28^ Second, the proportion of women with more than 1 first-degree relative with breast cancer was small (1.6% in discovery set; 0.3% in validation set), but we improved our power to find a statistically significant association between mammographic breast density and the FHBC in the large validation set. In addition, we did not have information on age at diagnosis for affected relatives. Studies suggested that the first-degree relative’s age at diagnosis varies the association between FHBC and the risk of breast cancer.^29,30^ Data from the Nurses’ Health Study indicates that women with a family member diagnosed with breast cancer before age 50 years of age had an increased risk of breast cancer compared with women with family members diagnosed at older ages.^30^ Our study population is mainly non-Hispanic White (discovery set: 65.9%, validation set: 57.1%), and non-Hispanic Black or African American (discovery set: 29.1%, validation set: 37.7%) with BMI in the overweight/obese categories, hence, our findings are likely to be generalizable to these groups. This is, however, representative of the population of the greater St. Louis region (mean BMI of 31.9; 73.4% are non-Hispanic White, and 18% are non-Hispanic Black),^31^ where most of our study participants come from. Lastly, within both the discovery and validation sets, the mean age was approximately 47 years; as a result, some of these women were closer to being perimenopausal.

## Conclusions

This cohort study found that having an FHBC was positively associated with mammographic breast density in premenopausal women, and the association was consistent and robust irrespective of whether qualitative or quantitative measures of mammographic breast density were used. Our findings indicate that women’s FHBC may play an important role in mammographic breast density, and underscore the need to begin annual screening mammogram at an early age in premenopausal women with an FHBC.

## Notes

Supplement. **eTable 1.** Associations of Family History of Breast Cancer With Dense Volume and Non-Dense Volume (N=375) **eTable 2.** Associations of Family History of Breast Cancer With BI-RADS Breast Density Categories 1 in Validation Set, Stratified by Race **eTable 3.** Associations of Family History of Breast Cancer With BI-RADS Breast Density Categories, Pre- and Post-5th Edition of BI-RADS
