# Supplementary material for: Family History of Breast Cancer and Mammographic Breast Density in Premenopausal Women
Source: JAMA Netw Open. Author manuscript; Available in PMC 2022 Apr 26. (PMC8855232; doi:10.1001/jamanetworkopen.2021.48983)
Supplement: Supplementary Material [file NIHMS1782753-supplement-Supplementary_Material.docx]

**Supplementary Online Content**

**eTable 1**. Associations of family history of breast cancer with dense volume and non-dense volume (N=375).

**eTable 2.** Associations of family history of breast cancer with BI-RADS breast density categories1 in validation set, stratified by race.

**eTable3.** Associations of family history of breast cancer with BI-RADS breast density categories, pre- and post-5th edition of BIRADS.

This supplementary material has been provided by the authors to give readers additional information about their work.

| **eTable 1.** Associations of family history of breast cancer with dense volume and non-dense volume (N=375). | | | | | |
| --- | --- | --- | --- | --- | --- |
| **Characteristics** | ***N*** | **Dense Volume (cm^3^)^a^** |  | **Non-dense Volume (cm^3^)^a^** | |
|  |  | **exp^β^ (95% CI)** | **p value^b^** | **exp^β^ (95% CI)** | **p value^b^** |
| **FHBC** |  |  |  |  |  |
| *Model 1*^c^ |  |  |  |  |  |
| No | 275 | Reference |  | Reference |  |
| Yes | 87 | 1.16 (1.05 – 1.29) | **0.006** | 0.91 (0.81 – 1.03) | 0.12 |
| Unknown | 13 | 0.79 (0.62 – 1.01) | 0.06 | 0.79 (0.60 – 1.03) | 0.09 |
| *Model 2*^d^ |  |  |  |  |  |
| No | 275 | Reference |  | Reference |  |
| Yes | 87 | 1.16 (1.04 – 1.29) | **0.007** | 0.91 (0.81 – 1.03) | 0.13 |
| Unknown | 13 | 0.74 (0.58 – 0.95) | **0.02** | 0.74 (0.56 – 0.98) | **0.04** |
| **FHBC in mothers alone** |  |  |  |  |  |
| *Model 1*^c^ |  |  |  |  |  |
| No | 301 | Reference |  | Reference |  |
| Yes | 67 | 1.21 (1.08 – 1.36) | **0.001** | 0.91 (0.80 – 1.04) | 0.15 |
| Unknown | 7 | 0.91 (0.66 – 1.27) | 0.59 | 0.88 (0.61 – 1.28) | 0.49 |
| *Model 2*^d^ |  |  |  |  |  |
| No | 301 | Reference |  | Reference |  |
| Yes | 67 | 1.21 (1.07 – 1.36) | **0.002** | 0.91 (0.79 – 1.04) | 0.15 |
| Unknown | 7 | 0.88 (0.63 – 1.22) | 0.44 | 0.84 (0.58 – 1.22) | 0.36 |
| **FHBC in sisters alone** |  |  |  |  |  |
| *Model 1*^c^ |  |  |  |  |  |
| No | 339 | Reference |  | Reference |  |
| Yes | 26 | 1.07 (0.90 – 1.28) | 0.45 | 0.98 (0.80 – 1.19) | 0.82 |
| Unknown | 10 | 0.75 (0.57 – 0.99) | **0.04** | 0.72 (0.53 – 0.98) | **0.04** |
| *Model 2*^d^ |  |  |  |  |  |
| No | 339 | Reference |  | Reference |  |
| Yes | 26 | 1.08 (0.91 – 1.29) | 0.38 | 0.99 (0.81 – 1.21) | 0.95 |
| Unknown | 10 | 0.71 (0.53 – 0.93) | **0.01** | 0.68 (0.50 – 0.93) | **0.02** |
| **Number of affected relatives with FHBC** |  |  |  |  |  |
| *Model 1*^c^ |  |  |  |  |  |
| 0 | 275 | Reference |  | Reference |  |
| 1 | 81 | 1.16 (1.04 – 1.29) | **0.01** | 0.99 (0.81 – 1.21) | 0.95 |
| ≥2 | 6 | 1.25 (0.87 – 1.78) | 0.23 | 0.68 (0.50 – 0.93) | **0.02** |
| Unknown | 13 | 0.79 (0.62 – 1.01) | 0.06 | 0.99 (0.81 – 1.21) | 0.95 |
| *Model 2*^d^ |  |  |  |  |  |
| 0 | 275 | Reference |  | Reference |  |
| 1 | 81 | 1.15 (1.03 – 1.28) | **0.01** | 0.91 (0.80 – 1.03) | 0.14 |
| ≥2 | 6 | 1.32 (0.92 – 1.89) | 0.13 | 0.91 (0.61 – 1.37) | 0.66 |
| Unknown | 13 | 0.74 (0.58 – 0.95) | **0.02** | 0.74 (0.56 – 0.98) | **0.037** |
| Abbreviations: CI, confidence interval, exp, Exponentiated; FHBC, Family history of breast cancer; N, Number.  ^a^ Continuous mammographic measurements were log transformed in the analysis. The beta coefficients were back transformed and exponentiated for easier interpretation. Effect measures represent proportion for percentage difference.  ^b^ p value derived from linear regression.  ^c^ Model 1 was adjusted for current age and body mass index (BMI)  ^d^ Model 2 was adjusted for current age, BMI, parity, race, age at menarche, and alcohol use. | | | | | |

| **eTable 2.** Associations of family history of breast cancer with BI-RADS breast density categories^a^ in validation set, stratified by race. | | | | | | | | |
| --- | --- | --- | --- | --- | --- | --- | --- | --- |
| **Characteristics** | **Non- Hispanic White**  **(N = 8,010)** | | | | **Black/African American**  **(N = 5,292)** | | | |
|  | **Dense breast**  **(N)** | **Total**  **(N)** | **Dense breast vs. non-dense breast** | | **Dense breast**  **(N)** | **Total**  **(N)** | **Dense breast vs. non-dense breast** | |
|  |  |  | **OR (95% CI)** | **p value^b^** |  |  | **OR (95% CI)** | **p value^b^** |
| **FHBC** |  |  |  |  |  |  |  |  |
| *Model 1*^c^ |  |  |  |  |  |  |  |  |
| No | 3464 | 6632 | Reference | **<0.001** | 1578 | 4572 | Reference | **0.03** |
| Yes | 787 | 1378 | 1.30 (1.13 – 1.49) |  | 279 | 720 | 1.23 (1.02 – 1.49) |  |
| *Model 2*^d^ |  |  |  |  |  |  |  |  |
| No | 3464 | 6632 | Reference | **<0.001** | 1578 | 4572 | Reference | **0.03** |
| Yes | 787 | 1378 | 1.29 (1.13 – 1.49) |  | 279 | 720 | 1.23 (1.02 – 1.49) |  |
| **FHBC in mothers alone** |  |  |  |  |  |  |  |  |
| *Model 1*^c^ |  |  |  |  |  |  |  |  |
| No | 3129 | 6059 | Reference | **<0.001** | 1371 | 4065 | Reference | **0.003** |
| Yes | 572 | 998 | 1.27 (1.09 – 1.49) |  | 173 | 448 | 1.25 (0.99 – 1.58) |  |
| Unknown | 550 | 953 | 1.36 (1.16 – 1.59) |  | 313 | 779 | 1.32 (1.10 – 1.57) |  |
| *Model 2*^d^ |  |  |  |  |  |  |  |  |
| No | 3129 | 6059 | Reference | **<0.001** | 1371 | 4065 | Reference | **0.003** |
| Yes | 572 | 998 | 1.27 (1.08 – 1.49) |  | 173 | 448 | 1.25 (0.99 – 1.58) |  |
| Unknown | 550 | 953 | 1.32 (1.12 – 1.55) |  | 313 | 779 | 1.34 (1.11 – 1.60) |  |
| **FHBC in sisters alone** |  |  |  |  |  |  |  |  |
| *Model 1*^c^ |  |  |  |  |  |  |  |  |
| No | 3126 | 6054 | Reference | **<0.001** | 1371 | 4064 | Reference | **0.003** |
| Yes | 150 | 284 | 1.36 (1.03 – 1.79) |  | 85 | 234 | 1.18 (0.87 – 1.62) |  |
| Unknown | 975 | 1672 | 1.31(1.15 – 1.49) |  | 401 | 994 | 1.32 (1.12 – 1.56) |  |
| *Model 2*^d^ |  |  |  |  |  |  |  |  |
| No | 3126 | 6054 | Reference | **0.002** | 1371 | 4064 | Reference | **0.003** |
| Yes | 150 | 284 | 1.37 (1.04 – 1.80) |  | 85 | 234 | 1.21 (0.88 – 1.66) |  |
| Unknown | 975 | 1672 | 1.28 (1.13 – 1.46) |  | 401 | 994 | 1.33 (1.13 – 1.57) |  |
| **Number of affected relatives with FHBC** |  |  |  |  |  |  |  |  |
| *Model 1*^c^ |  |  |  |  |  |  |  |  |
| 0 | 3125 | 6053 | Reference | **0.001** | 1369 | 4061 | Reference | **0.006** |
| 1 | 660 | 1168 | 1.30 (1.12 – 1.50) |  | 236 | 620 | 1.20 (0.98 – 1.47) |  |
| ≥2 | 31 | 57 | 1.27 (0.70 – 2.31) |  | 11 | 31 | 1.54 (0.68 – 3.49) |  |
| Unknown | 435 | 732 | 1.35 (1.13 – 1.62) |  | 241 | 580 | 1.38 (1.13 – 1.69) |  |
| *Model 2*^d^ |  |  |  |  |  |  |  |  |
| 0 | 3125 | 6053 | Reference | **0.006** | 1369 | 4061 | Reference | **0.005** |
| 1 | 660 | 1168 | 1.30 (1.12 – 1.50) |  | 236 | 620 | 1.21 (0.98 – 1.48) |  |
| ≥2 | 31 | 57 | 1.24 (0.68 – 2.26) |  | 11 | 31 | 1.56 (0.69 – 3.52) |  |
| Unknown | 435 | 732 | 1.30 (1.08 – 1.56) |  | 241 | 580 | 1.40 (1.14 – 1.73) |  |
| Abbreviations: BI-RADS, The Breast Imaging, Reporting and Data System; CI, confidence interval, FHBC, Family history of breast cancer; N, Number; OR, Odds ratio.  ^1^ Dense breast defined as BI-RADS 3 and 4. Non-dense breast (referent) defined as BI-RADS 1 and 2. Logistic regression examines odds of having dense breast.  ^2^ Type III chi-square p values derived from logistic regression.  ^3^ Model 1 was adjusted for current age and BMI  ^4^ Model 2 was adjusted for current age, BMI, parity, age at menarche, and alcohol use. | | | | | | | | |

| **eTable 3. Associations of family history of breast cancer with BI-RADS breast density categories, pre- and post-5^th^ edition of BIRADS.** | | | | | | |
| --- | --- | --- | --- | --- | --- | --- |
| **Characteristics** | **Visited Before December 31, 2012**  **(*N* = 6,900)** | | | **Visited After January 1, 2013**  **(*N* = 7,140)** | | |
|  | **Dense Breast (BI-RADS)^a^** | | | | | |
|  | ***N*^b^** | **Odds Ratio (95% CI)** | **p value^c^** | ***N*^b^** | **Odds Ratio (95% CI)** | **p value^c^** |
| **FHBC** |  |  |  |  |  |  |
| *Model 1*^d^ |  |  |  |  |  |  |
| No | 2717 | Reference | **0.02** | 2806 | Reference | **<0.001** |
| Yes | 469 | 1.22 (1.03 – 1.43) |  | 643 | 1.52 (1.16 – 1.55) |  |
| *Model 2*^e^ |  |  |  |  |  |  |
| No | 2717 | Reference | **0.01** | 2806 | Reference | **<0.001** |
| Yes | 469 | 1.23 (1.04 – 1.45) |  | 643 | 1.36 (1.17 – 1.58) |  |
| **FHBC in mothers alone** |  | 6 |  |  |  |  |
| *Model 1*^d^ |  |  |  |  |  |  |
| No | 2446 | Reference | **0.01** | 2476 | Reference | **<0.001** |
| Yes | 327 | 1.23 (1.02 – 1.50) |  | 441 | 1.30 (1.10 – 1.55) |  |
| Unknown | 413 | 1.25 (1.05 – 1.48) |  | 532 | 1.40 (1.19 – 1.63) |  |
| *Model 2*^e^ |  |  |  |  |  |  |
| No | 2446 | Reference | **0.01** | 2476 | Reference | **<0.001** |
| Yes | 327 | 1.24 (1.02 – 1.51) |  | 441 | 1.31 (1.10 – 1.56) |  |
| Unknown | 413 | 1.24 (1.04 – 1.47) |  | 532 | 1.39 (1.18 – 1.63) |  |
| **FHBC in sisters alone** |  |  |  |  |  |  |
| *Model 1*^d^ |  |  |  |  |  |  |
| No | 2442 | Reference | **<0.01** | 2477 | Reference | **<0.001** |
| Yes | 102 | 1.10 (0.82 – 1.48) |  | 154 | 1.55 (1.18 – 2.03) |  |
| Unknown | 642 | 1.28 (1.11 – 1.48) |  | 818 | 1.31 (1.15 – 1.50) |  |
| *Model 2*^e^ |  |  |  |  |  |  |
| No | 2442 | Reference | **<0.01** | 2477 | Reference | **<0.001** |
| Yes | 102 | 1.12 (0.83 – 1.52) |  | 154 | 1.58 (1.20 – 2.09) |  |
| Unknown | 642 | 1.27 (1.10 – 1.48) |  | 818 | 1.31 (1.14 – 1.50) |  |
| **Number of affected relatives with FHBC** |  |  |  |  |  |  |
| *Model 1*^d^ |  |  |  |  |  |  |
| 0 | 2442 | Reference | **0.01** | 2474 | Reference | **<0.001** |
| 1 | 403 | 1.22 (1.03 – 1.46) |  | 533 | 1.32 (1.13 – 1.55) |  |
| ≥2 | 13 | 0.91 (0.42 – 1.97) |  | 31 | 1.89 (0.99 – 3.60) |  |
| Unknown | 328 | 1.29 (1.07 – 1.57) |  | 411 | 1.36 (1.14 – 1.63) |  |
| *Model 2*^e^ |  |  |  |  |  |  |
| 0 | 2442 | Reference | **0.02** | 2474 | Reference | **<0.001** |
| 1 | 403 | 1.23 (1.03 – 1.47) |  | 533 | 1.34 (1.14 – 1.57) |  |
| ≥2 | 13 | 0.95 (0.44 – 2.06) |  | 31 | 1.83 (0.96 – 3.51) |  |
| Unknown | 328 | 1.27 (1.04 – 1.55) |  | 411 | 1.34 (1.12 – 1.61) |  |
| Abbreviations: BI-RADS, The Breast Imaging, Reporting and Data System; CI, confidence interval; exp, Exponentiated; FHBC, Family history of breast cancer; N, Number.  ^a^ Dense breast defined as BI-RADS 3 and 4. Non-dense breast (reference) defined as BI-RADS 1 and 2. Logistic regression examines odds of having dense breast.  ^b^ Represents the proportion of patients with dense breast within the exposure strata (i.e., among those with or without a family history of breast cancer).  ^c^ Type III chi-square p values derived from logistic regression.  ^d^ Model 1 was adjusted for current age (continuous, years) and BMI (continuous, kg/m^2^).  ^e^ Model 2 was adjusted for current age (continuous, years), BMI (continuous, kg/m^2^), parity (0, 1, 2, ≥3), race (non-Hispanic white/African American/others), age at menarche (continuous, years), and alcohol consumption (yes/no). | | | | | | |
