# Supplementary material for: Family History of Breast Cancer and Mammographic Breast Density in Premenopausal Women
Source: JAMA Netw Open. 2022 Feb 17;5(2):e2148983. doi: 10.1001/jamanetworkopen.2021.48983 (PMC8855232; doi:10.1001/jamanetworkopen.2021.48983)
Supplement: Supplementary file 1 [file jamanetwopen-e2148983-s001.pdf]

Supplemental Online Content

Han Y, Moore JX, Colditz GA, Toriola AT. Family history of breast cancer and mammographic breast density in premenopausal women. *JAMA Netw Open*. 2022;5(2):e2148983. doi:10.1001/jamanetworkopen.2021.48983

eTable 1. Associations of Family History of Breast Cancer With Dense Volume and Non-Dense Volume (N=375)

eTable 2. Associations of Family History of Breast Cancer With BI-RADS Breast Density Categories 1 in Validation Set, Stratified by Race

eTable 3. Associations of Family History of Breast Cancer With BI-RADS Breast Density Categories, Pre- and Post-5th Edition of BI-RADS

This supplementary material has been provided by the authors to give readers additional information about their work.

© 2022 Han Y et al. *JAMA Network Open.*

**eTable 1.** Associations of family history of breast cancer with dense volume and non-dense volume (N=375).					
**Characteristics**	***N***	**Dense Volume (cm^3)[a]**		**Non-dense Volume (cm^3)[a]**	
		**exp$^\beta$ (95% CI)**	**p value[b]**	**exp$^\beta$ (95% CI)**	**p value[b]**
**FHBC**					
Model 1[c]					
No	275	Reference		Reference	
Yes	87	1.16 (1.05 – 1.29)	**0.006**	0.91 (0.81 – 1.03)	0.12
Unknown	13	0.79 (0.62 – 1.01)	0.06	0.79 (0.60 – 1.03)	0.09
Model 2[d]					
No	275	Reference		Reference	
Yes	87	1.16 (1.04 – 1.29)	**0.007**	0.91 (0.81 – 1.03)	0.13
Unknown	13	0.74 (0.58 – 0.95)	**0.02**	0.74 (0.56 – 0.98)	**0.04**
**FHBC in mothers alone**					
Model 1[c]					
No	301	Reference		Reference	
Yes	67	1.21 (1.08 – 1.36)	**0.001**	0.91 (0.80 – 1.04)	0.15
Unknown	7	0.91 (0.66 – 1.27)	0.59	0.88 (0.61 – 1.28)	0.49
Model 2[d]					
No	301	Reference		Reference	
Yes	67	1.21 (1.07 – 1.36)	**0.002**	0.91 (0.79 – 1.04)	0.15
Unknown	7	0.88 (0.63 – 1.22)	0.44	0.84 (0.58 – 1.22)	0.36
**FHBC in sisters alone**					
Model 1[c]					
No	339	Reference		Reference	
Yes	26	1.07 (0.90 – 1.28)	0.45	0.98 (0.80 – 1.19)	0.82
Unknown	10	0.75 (0.57 – 0.99)	**0.04**	0.72 (0.53 – 0.98)	**0.04**
Model 2[d]					
No	339	Reference		Reference	
Yes	26	1.08 (0.91 – 1.29)	0.38	0.99 (0.81 – 1.21)	0.95
Unknown	10	0.71 (0.53 – 0.93)	**0.01**	0.68 (0.50 – 0.93)	**0.02**
**Number of affected relatives with FHBC**					
Model 1[c]					
0	275	Reference		Reference	
1	81	1.16 (1.04 – 1.29)	**0.01**	0.99 (0.81 – 1.21)	0.95
≥2	6	1.25 (0.87 – 1.78)	0.23	0.68 (0.50 – 0.93)	**0.02**
Unknown	13	0.79 (0.62 – 1.01)	0.06	0.99 (0.81 – 1.21)	0.95
Model 2[d]					
0	275	Reference		Reference	
1	81	1.15 (1.03 – 1.28)	**0.01**	0.91 (0.80 – 1.03)	0.14
≥2	6	1.32 (0.92 – 1.89)	0.13	0.91 (0.61 – 1.37)	0.66
Unknown	13	0.74 (0.58 – 0.95)	**0.02**	0.74 (0.56 – 0.98)	**0.037**

Abbreviations: CI, confidence interval, exp, Exponentiated; FHBC, Family history of breast cancer; N, Number.

[a] Continuous mammographic measurements were log transformed in the analysis. The beta coefficients were back transformed and exponentiated for easier interpretation. Effect measures represent proportion for percentage difference.

[b] p value derived from linear regression.

[c] Model 1 was adjusted for current age and body mass index (BMI)

[d] Model 2 was adjusted for current age, BMI, parity, race, age at menarche, and alcohol use.

© 2022 Han Y et al. *JAMA Network Open.*

eTable 2. Associations of family history of breast cancer with BI-RADS breast density categories[a] in validation set, stratified by race.

**Characteristics**	**Non- Hispanic White (N = 8,010)**				**Black/African American (N = 5,292)**			
	**Dense breast (N)**	**Total (N)**	**Dense breast vs. non-dense breast**		**Dense breast (N)**	**Total (N)**	**Dense breast vs. non-dense breast**	
			**OR (95% CI)**	**p value[b]**			**OR (95% CI)**	**p value[b]**
**FHBC**								
Model 1[c]								
No	3464	6632	Reference	**<0.001**	1578	4572	Reference	**0.03**
Yes	787	1378	1.30 (1.13 – 1.49)		279	720	1.23 (1.02 – 1.49)	
Model 2[d]								
No	3464	6632	Reference	**<0.001**	1578	4572	Reference	**0.03**
Yes	787	1378	1.29 (1.13 – 1.49)		279	720	1.23 (1.02 – 1.49)	
**FHBC in mothers alone**								
Model 1[c]								
No	3129	6059	Reference	**<0.001**	1371	4065	Reference	**0.003**
Yes	572	998	1.27 (1.09 – 1.49)		173	448	1.25 (0.99 – 1.58)	
Unknown	550	953	1.36 (1.16 – 1.59)		313	779	1.32 (1.10 – 1.57)	
Model 2[d]								
No	3129	6059	Reference	**<0.001**	1371	4065	Reference	**0.003**
Yes	572	998	1.27 (1.08 – 1.49)		173	448	1.25 (0.99 – 1.58)	
Unknown	550	953	1.32 (1.12 – 1.55)		313	779	1.34 (1.11 – 1.60)	
**FHBC in sisters alone**								
Model 1[c]								
No	3126	6054	Reference	**<0.001**	1371	4064	Reference	**0.003**
Yes	150	284	1.36 (1.03 – 1.79)		85	234	1.18 (0.87 – 1.62)	
Unknown	975	1672	1.31(1.15 – 1.49)		401	994	1.32 (1.12 – 1.56)	
Model 2[d]								
No	3126	6054	Reference	**0.002**	1371	4064	Reference	**0.003**
Yes	150	284	1.37 (1.04 – 1.80)		85	234	1.21 (0.88 – 1.66)	
Unknown	975	1672	1.28 (1.13 – 1.46)		401	994	1.33 (1.13 – 1.57)	
**Number of affected relatives with FHBC**								
Model 1[c]								
0	3125	6053	Reference	**0.001**	1369	4061	Reference	**0.006**
1	660	1168	1.30 (1.12 – 1.50)		236	620	1.20 (0.98 – 1.47)	
≥2	31	57	1.27 (0.70 – 2.31)		11	31	1.54 (0.68 – 3.49)	
Unknown	435	732	1.35 (1.13 – 1.62)		241	580	1.38 (1.13 – 1.69)	
Model 2[d]								
0	3125	6053	Reference	**0.006**	1369	4061	Reference	**0.005**
1	660	1168	1.30 (1.12 – 1.50)		236	620	1.21 (0.98 – 1.48)	
≥2	31	57	1.24 (0.68 – 2.26)		11	31	1.56 (0.69 – 3.52)	
Unknown	435	732	1.30 (1.08 – 1.56)		241	580	1.40 (1.14 – 1.73)	

Abbreviations: BI-RADS, The Breast Imaging, Reporting and Data System; CI, confidence interval, FHBC, Family history of breast cancer; N, Number; OR, Odds ratio.
[1] Dense breast defined as BI-RADS 3 and 4. Non-dense breast (referent) defined as BI-RADS 1 and 2. Logistic regression examines odds of having dense breast.
[2] Type III chi-square p values derived from logistic regression.
[3] Model 1 was adjusted for current age and BMI
[4] Model 2 was adjusted for current age, BMI, parity, age at menarche, and alcohol use.

© 2022 Han Y et al. *JAMA Network Open.*

eTable 3. Associations of family history of breast cancer with BI-RADS breast density categories, pre- and post-5th edition of BIRADS.

**Characteristics**	**Visited Before December 31, 2012 (*N* = 6,900)**			**Visited After January 1, 2013 (*N* = 7,140)**		
	**Dense Breast (BI-RADS)[a]**					
	***N*[b]**	**Odds Ratio (95% CI)**	**p value[c]**	***N*[b]**	**Odds Ratio (95% CI)**	**p value[c]**
**FHBC**						
Model 1[d]						
No	2717	Reference	**0.02**	2806	Reference	**<0.001**
Yes	469	1.22 (1.03 – 1.43)		643	1.52 (1.16 – 1.55)	
Model 2[e]						
No	2717	Reference	**0.01**	2806	Reference	**<0.001**
Yes	469	1.23 (1.04 – 1.45)		643	1.36 (1.17 – 1.58)	
**FHBC in mothers alone**		6				
Model 1[d]						
No	2446	Reference	**0.01**	2476	Reference	**<0.001**
Yes	327	1.23 (1.02 – 1.50)		441	1.30 (1.10 – 1.55)	
Unknown	413	1.25 (1.05 – 1.48)		532	1.40 (1.19 – 1.63)	
Model 2[e]						
No	2446	Reference	**0.01**	2476	Reference	**<0.001**
Yes	327	1.24 (1.02 – 1.51)		441	1.31 (1.10 – 1.56)	
Unknown	413	1.24 (1.04 – 1.47)		532	1.39 (1.18 – 1.63)	
**FHBC in sisters alone**						
Model 1[d]						
No	2442	Reference	**<0.01**	2477	Reference	**<0.001**
Yes	102	1.10 (0.82 – 1.48)		154	1.55 (1.18 – 2.03)	
Unknown	642	1.28 (1.11 – 1.48)		818	1.31 (1.15 – 1.50)	
Model 2[e]						
No	2442	Reference	**<0.01**	2477	Reference	**<0.001**
Yes	102	1.12 (0.83 – 1.52)		154	1.58 (1.20 – 2.09)	
Unknown	642	1.27 (1.10 – 1.48)		818	1.31 (1.14 – 1.50)	
**Number of affected relatives with FHBC**						
Model 1[d]						
0	2442	Reference	**0.01**	2474	Reference	**<0.001**
1	403	1.22 (1.03 – 1.46)		533	1.32 (1.13 – 1.55)	
≥2	13	0.91 (0.42 – 1.97)		31	1.89 (0.99 – 3.60)	
Unknown	328	1.29 (1.07 – 1.57)		411	1.36 (1.14 – 1.63)	
Model 2[e]						
0	2442	Reference	**0.02**	2474	Reference	**<0.001**
1	403	1.23 (1.03 – 1.47)		533	1.34 (1.14 – 1.57)	
≥2	13	0.95 (0.44 – 2.06)		31	1.83 (0.96 – 3.51)	
Unknown	328	1.27 (1.04 – 1.55)		411	1.34 (1.12 – 1.61)	

Abbreviations: BI-RADS, The Breast Imaging, Reporting and Data System; CI, confidence interval; exp, Exponentiated; FHBC, Family history of breast cancer; N, Number.

[a] Dense breast defined as BI-RADS 3 and 4. Non-dense breast (reference) defined as BI-RADS 1 and 2. Logistic regression examines odds of having dense breast.

[b] Represents the proportion of patients with dense breast within the exposure strata (i.e., among those with or without a family history of breast cancer).

[c] Type III chi-square p values derived from logistic regression.

[d] Model 1 was adjusted for current age (continuous, years) and BMI (continuous, kg/m^2).

[e] Model 2 was adjusted for current age (continuous, years), BMI (continuous, kg/m^2), parity (0, 1, 2, ≥3), race (non-Hispanic white/African American/others), age at menarche (continuous, years), and alcohol consumption (yes/no).

© 2022 Han Y et al. *JAMA Network Open.*
